# Long-Term Survivor with Paraneoplastic Cerebellar Ataxia and Small-Cell Lung Cancer

**DOI:** 10.3390/jcm14020364

**Published:** 2025-01-09

**Authors:** Konstantinos Tsoukalas, Ioannis Ntanasis-Stathopoulos, Angeliki Andrikopoulou, John S. Tzartos, Meletios A. Dimopoulos, Maria Gavriatopoulou

**Affiliations:** 1 Department of Clinical Therapeutics, Alexandra General Hospital, School of Medicine, National and Kapodistrian University of Athens, 11528 Athens, Greece; ktsoukalas@med.uoa.gr (K.T.);; 2 2nd Department of Neurology, “Attikon” University Hospital, School of Medicine, National and Kapodistrian University of Athens, 12462 Athens, Greece; 3 Department of Medicine, Korea University, Seoul 02841, Republic of Korea

**Keywords:** paraneoplastic neurological syndrome, paraneoplastic cerebellar degradation, small-cell lung cancer, onconeural antibodies, anti-CV2, anti-SOX1

## Abstract

**Background/Objectives:** Paraneoplastic cerebellar degeneration (PCD) is an inflammatory autoimmune process caused by onconeural antibodies directed against cerebellar Purkinje cells. In most cases, prognosis is poor as disease progression leads to pancerebellar dysfunction and permanent neurological damage. Through this case report, we aim to highlight the clinical presentation, diagnostic process, and therapeutic implications associated with PCD secondary to SCLC. **Methods:** Herein, we present the case of a 57-year-old patient diagnosed with PCD who presented with progressive limb ataxia and impaired mobility. CT scans and EBUS (endobronchial ultrasound) bronchoscopy established the diagnosis of limited-stage small-cell lung cancer (SCLC) of the right lung with marked lymphadenopathy. **Results:** Anti-CV2/CRMP5 and anti-SOX1 autoantibodies were identified in the serum that confirmed the diagnosis of PCD related to SCLC. A total of six cycles of chemotherapy with carboplatin and etoposide resulted in rapid clinical improvement and complete response of the disease. The patient remains in remission six years after the initial diagnosis with no neurological deficits. **Conclusions:** The prognosis of PCD greatly depends on early detection and management of the underlying malignancy. Despite the poor prognosis, early diagnosis and prompt initiation of chemotherapy may offer a great survival benefit in these patients.

## 1. Introduction

Paraneoplastic cerebellar degeneration (PCD) is a rare paraneoplastic neurological syndrome (PNS) encountered in less than 1% of patients with cancer [1]. PCD is an inflammatory autoimmune process caused by onconeural antibodies directed against cerebellar Purkinje cells [1]. The first case of PCD was reported from Brouwer back in 1919 in a patient with pelvic sarcoma. Since then, several other cases have been reported, mainly in patients diagnosed with gynecologic and breast cancer, but also small-cell lung cancer (SCLC) and Hodgkin disease [2,3,4]. PCD is characterized by acute or subacute progressive pancerebellar dysfunction presenting with limb and truncal ataxia, dysarthria, and nystagmus [5]. Cerebellar degeneration may precede cancer diagnosis by months to years. Only 50% of patients with suspected PCD are positive for antineuronal antibodies in serum or cerebrospinal fluid (CSF) [6]. Several onconeural antibodies have been identified in patients with PCD. These antibodies are produced by the immune system in response to antigens expressed by cancer cells, and they are able to recognize a specific epitope on the Purkinje cells in the cerebellum [1]. SCLC is associated with multiple antibodies, including anti-Hu, voltage-gated calcium channel antibodies (VGCC), and antibodies against CRMP-5 [6]. Overall, the prognosis of PCD is poor, and early detection and treatment are crucial.

We here report a case of PCD diagnosed in a patient with SCLC that was successfully treated. We also present a review of the literature on this rare clinical entity to better characterize the clinical features and prognosis.

## 2. Case Report

A 57-year-old Caucasian woman presented initially to the neurology emergency department with a one-month history of progressive ataxia in her limbs that severely impaired her mobility. The patient also reported weight loss (7 kg in the last two months, more than 10% of her baseline total body weight). She had a recent history of gastrointestinal infection. No headaches, diplopia, cognition, or language disorders were noted. Her past medical history included hypertension under treatment, Graves disease, and vitiligo, whereas she had undergone total hysterectomy with bilateral salpingo-oophorectomy due to diffuse uterine leiomyomatosis. Furthermore, she was an active smoker for 20 pack-years. Τhe patient was alert and well oriented in time and space with a Glasgow Coma Scale (GCS) score of 15/15. Vital signs at the emergency department included a temperature of 36.5 °C, a heart rate of 86 beats per minute, blood pressure measuring 118/69 mmHg, a respiratory rate of 16 breaths per minute, and an oxygen saturation of 95% on ambient air.

Neurological examination revealed an intact level of consciousness with limited dorsiflexion, hip flexion, and rotation bilaterally. The patient had an ataxic gait with a wide base of support, balance impairment, and defective interlimb coordination, which was suggestive of cerebellar gait ataxia. The ECOG performance status (PS) was 4, as the patient was confined to a bed or chair. Routine laboratory workup at admission was unremarkable. There were no signs or symptoms suggestive of infection.

**Figure 1:**
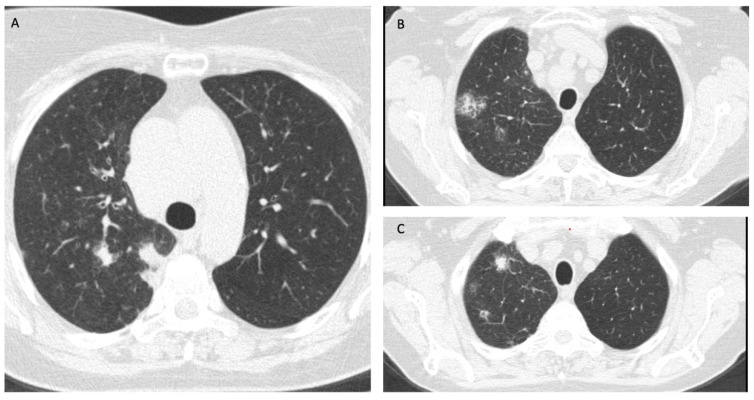
(**A**–**C**) Multifocal deposits of SCLC in the right lung at diagnosis.

Magnetic resonance imaging (MRI) of the brain on admission showed no signs of an invasive lesion or other abnormal findings. A computed tomography (CT) scan of the chest, abdomen, and pelvis showed multiple solid lesions on the right lung (especially the upper part of the inferior lobe, the apex, and the hilum of the right lung) with surrounding ground glass regions, mediastinal and hilar lymphadenopathy (Figure 1). EBUS (endobronchial ultrasound) bronchoscopy was performed, and a biopsy of the right hilar lymph node was taken. The pathology report revealed small-sized malignant cells with scant cytoplasm staining positive for thyroid transcription factor-1 (TTF-1) and synaptophysin, which was indicative of SCLC. The Ki67 index was approximately 85%. There was evidence of extensive crush artifact and prominent necrosis. Patchy lymphocytic infiltration was evident within the tumor nests themselves, though less dense than at the periphery. Therefore, the diagnosis of limited-stage SCLC was established.

Taking all the above into consideration, we suspected the clinical diagnosis of PCD associated with SCLC. We examined a panel of serum paraneoplastic antibodies that were strongly positive for anti-CV2/CRMP5 and anti-SOX1 onconeural antibodies, whereas anti-amphiphysine, anti-PNMA2 (Ma2/Ta), anti-Ri/ANNA-2, anti-Yo/PCA-1, and anti-Hu/ANNA-1 antibodies were negative. Cerebrospinal fluid (CSF) was clear and colorless, whereas further examination showed lymphocytic pleocytosis (leukocytes 33/mm^3^, 100% lymphocytes), increased protein levels (92 mg/dL), normal glucose levels (73 mg/dL, CSF to serum glucose ratio 0.54), no oligoclonal bands, and no signs of central nervous system infiltration, as the cytology was negative for malignant cells.

According to the revised criteria for paraneoplastic neurological syndromes by the European PNS Database, we reached the diagnosis of PCD based on the ongoing tumor history, the typical clinical attributes, and the onconeural antibody positivity [7].

The patient was started on chemotherapy with carboplatin (AUC 5) and etoposide (100 mg/m^2^) every 21 days. At that time, immunotherapy was not approved in the first-line setting of SCLC. The patient showed gradual clinical improvement after the second cycle of chemotherapy, with improvements in balance and gait. The patient completed six cycles of chemotherapy with no significant complications or adverse effects. Restaging with CT scans showed a complete response in the lesions of the right lung. Subsequently, the patient underwent consolidative thoracic radiation (total dose of 30 Gy) and prophylactic cranial irradiation (PCI). Restaging post-radiotherapy with FDG positron emission tomography (PET)/CT scan showed no evidence of disease progression or recurrence. The patient received no further treatment, and her clinical condition showed further improvement with a PS of 1 at the one-year follow-up.

At 5.5 years post-diagnosis, we re-evaluated the levels of serum paraneoplastic antibodies, and we showed a persistent expression of anti-CV2/CRMP5 onconeural antibodies at a titer of 61 (margins of positivity 51–150, based on Western Blot analysis). Currently, the patient remains in remission at 6 years after the initial diagnosis with no relapse evident during the CT scans. The patient has a PS ECOG of 0 and she walks independently without the need of additional support.

## 3. Discussion

Paraneoplastic syndromes are a group of clinical disorders that occur from tissue or organ damage in areas remote from the original tumor and are fueled by tumor-derived or tumor-defense mechanisms. Although this case depicts a rare but clinically significant autoimmune-mediated paraneoplastic disorder, it is important to note that paraneoplastic syndromes are also caused by ectopic production of endocrine, metabolic, or other factors. Paraneoplastic syndromes are diagnosed in about 1–7.4% of patients with cancer [8]. The most common malignancy related to paraneoplastic syndromes is lung cancer, especially small-cell lung cancer (SCLC) [8]. Paraneoplastic neurological syndrome (PNS) is an immune-mediated neurological disorder related to cancer that is caused by indirect and remote effects of the primary tumor on the nervous system. Initially, it was estimated that PNS may affect about 1 in 10,000 patients with cancer; however, more recent population-based studies indicate a 30-fold increased prevalence that has reached 1 in 300 patients with cancer [7]. An estimated 3–5% of cases of SCLC will develop PNS [8]. PNS can affect all parts of the nervous system. There are three major types of PNS: (a) those that involve the central nervous system, such as cerebellar degeneration and limbic encephalitis; (b) those that involve the peripheral nervous system, e.g., sensory neuropathy; and (c) those that are mostly muscle-directed, such as dermatomyositis. Solid tumors, especially SCLC, breast, ovarian, and non-small-cell lung cancer (NSCLC), are the malignancies most commonly associated with PNS, accounting for over 60% of the cases. Hematologic diseases are less frequent, with PNS occurring in a minority of patients with Hodgkin lymphoma.

PCD is the second most frequent paraneoplastic presentation and is mediated by an impaired immunologic response against Purkinje cells in the cerebellum [1]. PCD is an inflammatory autoimmune process in response to tumor antigens that can be intracellular, cell-surface, or synaptic. An association between PCD and gynecologic cancers (breast or ovarian) was first identified in 1938, and subsequently the syndrome was well characterized by Brain in 1951 [9]. PCD occurs predominantly in patients with gynecological cancer, breast cancer, SCLC, or Hodgkin lymphoma. In one study, PCD was observed in 28% of PNS, occurring in 2 out of every 1000 patients with cancer [10].

Most PCD cases develop sub-acutely over a few weeks or months [1]. Disease typically begins with mild symptoms resembling a viral-like illness with dizziness, malaise, nausea, and vomiting. These symptoms progress acutely or subacutely to gait unsteadiness followed by ataxia and diplopia [1]. Oscillopsia, dysarthria, tremor, and sometimes dysphagia and blurry vision may be present. The initiation of a cerebellar syndrome may precede cancer diagnosis by a substantial amount of time—even years [1]. The clinical deterioration observed in PCD may vary; however, symptoms usually progress gradually over weeks to months [1]. Severe impairment leaving the patient in a disabled condition is the typical outcome [1]. Prognosis may depend on the type of the underlying malignancy. For example, patients with PCD associated with breast cancer tend to live longer compared to patients with gynecologic cancer (median survival 100 vs. 22 months) [11]. In addition, patients positive for anti-Yo antibodies and anti-Hu antibodies tend to present with more refractory disease and they have dismal prognosis compared to patients with anti-Tr antibodies and anti-Ri-antibodies [1]. Neither cancer stage at diagnosis nor survival correlates with neurological manifestations or autoantibody positivity [12]. The only predictor of survival in patients with PCD and SCLC is stage disease at diagnosis [12]. Similarly, our patient was diagnosed with limited-stage SCLC, despite the major neurological manifestations. The prompt initiation of anti-cancer treatment and the consolidative strategy with radiotherapy resulted in complete tumor response and long-term remission of both the PCD and the underlying malignancy.

The diagnosis of PNS requires the exclusion of other conditions related to the underlying malignancy, such as infections and coagulopathy disorders that could lead to neurological deficits. The presence of autoantibodies in the patient serum and/or CSF, along with an inflammatory image of the CSF consisting of pleocytosis or oligoclonal IgG bands, are strongly indicative of PNS. A consensus panel by Graus et al. presented the criteria for “definite”, “probable”, and “possible” PNS [7]. This diagnosis is based on a scoring system (PNS-Care Score) that combines the type of clinical phenotype, presence or absence of neuronal antibodies, and presence or absence of cancer. Onconeural antibodies are also divided into “high-risk antibodies”, which are associated very frequently (>70%) with cancer, and “intermediate risk” (30–70% of cases) and “lower-risk” antibodies, which have a low (<30%) or absent association with cancer (Table 1).

The high-risk antibodies include anti-Hu, anti-Yo (PCA-1), anti-Tr (DNER), anti-CV2/CRMP5, anti-amphiphysin, anti-Ri (ANNA-2), anti-Ma2, and anti-SOX1. There are also antibodies described in the literature that target neuronal antigens but have a weak correlation with malignancies (e.g., NMDAR and GABAb receptors). Many PNS, like cerebellar degradation, are linked to certain antibodies, which suggests that these conditions are triggered by the immune system. The exact mechanism is not well established, but since the targeted antigens are mostly intracellular, it is considered unlikely that the antibodies are directly cytotoxic. The fact that the immediate removal of these antibodies from the serum via plasmapheresis does not appear to improve the clinical situation of the affected patients supports this observation. Thus, it is thought that cytotoxic T-cells trigger a degeneration of the Purkinje cells in the cerebellum, whereas onconeuronal antibodies may serve as predictive and diagnostic markers.

In patients with PCD, several onconeural antibodies have been detected, including anti-Hu, anti-Yo, anti-CV2, anti-Ma2, and anti-Tr. Other antibodies that have been detected include anti-glutamic acid decarboxylase (GAD) 65, anti-SRY-related HMG-box gene 1 (SOX-1), and anti-voltage-gated calcium channel (VGCC) antibodies [13]. More than 90% of patients with SCLC and neurological disorder are seropositive for at least one onconeural autoantibody [12]. In addition, onconeural autoantibodies are detected in 40% of patients with SCLC without a clinically evident neurological syndrome [12]. One antibody can be associated with different syndromes, while one syndrome may be related to different antibodies.

The antibodies are mostly specific to the type of tumor rather than the syndrome itself, which may seem helpful in narrowing down the investigation for malignancy. For example, a typical anti-Yo-positive patient is a postmenopausal woman with gynecological cancer. PCD associated with strong positivity of anti-Yo antibodies tends to have the worst prognosis.

Patients with Hu antibodies are more frequently diagnosed with SCLC than those with anti-Yo and have the same frequency in males and females [14]. Anti-Hu antibodies are a highly specific indicator for the presence of SCLC, as 70–85% of patients with anti-Hu antibodies are diagnosed with SCLC at some point [15]. Detectable levels of serum anti-Hu antibodies may be found in ~20% of patients with SCLC, although not all will develop a neurological disorder [16,17]. The Hu antigens are intracellular proteins, normally expressed throughout the central and peripheral nervous system. Anti-Hu antibodies are also related to extracerebellar symptoms, including dementia, muscular weakness, dysphagia, nystagmus, and abnormal reflexes. Approximately 13–20% of patients with Hu antibodies present with a subacute cerebellar syndrome that cannot initially be differentiated from PCD [14]. The presence of anti-Hu antibodies appears to be protective and is associated with increased tumor chemosensitivity, indolent tumor growth, response to therapy, and prolonged survival [17].

Anti-Sry-like high mobility group box 1 (antiSOX1) antibodies are partially characterized onconeural antibodies that target SOX1 transcription factors that contribute significantly to the development of the central nervous system. About 18.2% of patients diagnosed with PCD are positive for anti-SOX-1 antibodies [13]. SOX protein is also implicated in the differentiation of airway epithelial cells and SCLC carcinogenesis [18]. Anti-SOX-1 antibodies cross-react with the nuclei of cerebellar Bergmann glial cells and induce cerebellar ataxia [18]. These autoantibodies are also a specific predictor of SCLC in PCD with a high specificity of almost 100% [13].

Another type of “high-risk autoantibodies” is anti-CV2/CRMP5. The CV2/collapsin response-mediator protein-5 (CRMP) belongs to a family of proteins expressed both in the central nervous system, and in the peripheral nervous system where it contributes to the life cycle of the Schwann cells and the neural axon repair. Detection of anti-CV2/CRMP5 is rare, as only 7% of patients with PCD are positive for these antibodies [19]. Anti-CV2/CRMP5 antibodies are most often associated with SCLC and thymoma, whereas the most common clinical manifestations include peripheral neuropathy, uveitis, and cerebral ataxia, the latter of which was reported in our case [19]. Neuropathy is observed in 60% of patients with PCD and CV2/CRMP5 antibodies [14]. Other clinical features include cerebellar ataxia (41%), myelopathy (30%), myasthenia gravis (26%), autoimmune encephalopathy (26%), and optic neuropathy (22%) [19]. Patients with CV2/CRMP5 antibodies are mostly men (70%), and the most frequently associated tumor is SCLC (60%), as in our case. Although dysautonomia and peripheral neuropathy are more common in anti-Hu PNS, they have also been related to anti-CV2/CRMP5 PNS [15]. Motor involvement in anti-Hu or anti-CV2/CRMP5 PNS is uncommon and usually exists as part of a sensorimotor neuropathy with predominant sensory symptoms and only mild motor impairment [15]. In our case, the long-term persistence of anti-CV2 antibodies may be the result of persistent memory B-cells. This finding confirms that the value of assessing onconeural antibodies lies primarily in the differential diagnosis rather than in the monitoring of disease status. Patients who are free of cancer after treatment but present persistent positivity in onconeural antibodies may have an “immunological scarring” as a result of their previous immunologic encounter with cancer. Prospective studies with sequential evaluations of the presence and the titer of onconeural antibodies in patients with cancer and paraneoplastic syndromes are needed to elucidate the kinetics and the potential clinical significance.

Tr antibodies are usually detected in patients with PCD and Hodgkin’s disease, which is the third most common cancer associated with PCD, after SCLC and ovarian cancer [14]. Unlike other antibodies, anti-Tr usually disappears after treatment of the tumor or, in a few patients, is only found in the CSF [14]. A reappearance or an increase in the titer of onconeural antibodies, especially in combination with deteriorating clinical presentation, may be an early sign of relapsing malignancy, even before the imaging manifestation of the relapse. Of note, treatment with immune checkpoint inhibitors may enhance the immune response and thus facilitate or increase paraneoplastic neurologic symptoms [12,19].

Brain MRI imaging is initially normal but can demonstrate cerebellar atrophy in the latter stages of the disease [14]. Indeed, cerebellar atrophy is usually delayed and occurs months after the establishment of neurological symptoms [8]. However, CT and MRI imaging of the brain should be performed to rule out vascular events or malignant CNS involvement in suspected PCD cases. In patients with PCD and no detected antibodies and no other profound etiology of neurological impairment, FDG-PET SCAN should be performed to complete the necessary workup in the differential diagnosis, especially in case of an occult malignancy. Pathological examination may reveal loss of Purkinje cells and thinning of the molecular and granular layers of the cerebellum along with degeneration of the dentate nuclei, olivary nuclei, and long tracts of the spinal cord [8]. CSF examination shows inflammation without cancer cells (e.g., pleocytosis and oligoclonal bands) in about 60% of the cases. Mild pleocytosis, protein elevation, and/or oligoclonal bands are the typical CSF findings, although they are neither sensitive nor specific [1]. In this context, it is important to highlight the need for a multidisciplinary approach in patients with suspected paraneoplastic syndromes. This is especially relevant in cases where a neurological manifestation is the presenting symptom of an underlying undiagnosed cancer, as in our case. The early oncologic evaluation and the integration of neurologic and oncologic expertise are essential to guide the differential diagnosis, complete the workup timely, make a prompt diagnosis, and improve patient outcomes.

Early detection and treatment of the underlying tumor is the optimal treatment for PCD [14]. Immunotherapy is rarely effective, but there have been reports of an improvement in a few patients after the administration of intravenous immunoglobulin, steroids, or plasmapheresis. Patients with anti-Tr and Hodgkin disease have generally better prognosis than those with other antibodies [8,14]. In patients with Yo antibodies, the prognosis is worse in patients with ovarian cancer and better in patients with breast cancer. The absence of onconeural antibodies is also associated with a more favorable prognosis compared to patients with anti-Hu antibodies.

## 4. Conclusions

In the aforementioned case, we present a long-term SCLC survivor with PCD and persistently positive onconeural antibodies. Taking into consideration the clinical course of our patient, the clinical utility of the onconeural antibodies may lie primarily in the diagnosis rather than in the surveillance of patients with PCD. Determining the clinical relevance of persistent onconeural antibodies in patients in remission and their potential role in long-term surveillance or prognostication necessitates prospective studies in the field. Given its low frequency, it is crucial that clinicians have a high clinical suspicion to identify this clinical entity promptly in order to improve patient outcomes. A multidisciplinary approach is essential for the differential diagnosis of patients presenting with symptoms that cannot be attributed to an obvious underlying cause, as these may point to a paraneoplastic syndrome secondary to an occult malignancy. This is particularly important for patients who are considered at high risk for developing cancer due to genetic predisposition and/or exposure to predisposing factors, such as smoking and alcohol.

## Figures and Tables

**Table 1:** Autoantibodies with high and intermediate cancer association: prevalence, cancer types, and symptoms.

Autoantibody	Prevalence of Cancer (%)	Associated Cancer Types	Neurological Symptoms/Syndromes
Hu (ANNA-1)	>85	SCLC, neuroendocrine tumors	SNN, chronic gastrointestinal pseudo-obstruction, EM, LE
CV2/CRMP5	>80	SCLC, thymoma	EM, SNN, myasthenia gravis
SOX1	>90	SCLC	LEMS, rapidly progressive cerebellar syndrome
PCA2 (MAP1B)	80	SCLC, NSCLC, and breast cancer	Sensorimotor neuropathy, rapidly progressive cerebellar syndrome, and EM
Amphiphysin	80	SCLC and breast cancer	Polyradiculoneuropathy, SNN, EM, SPS
Ri (ANNA-2)	>70	Breast cancer, SCLC	OMS, brainstem or cerebellar syndrome
Yo (PCA-1)	>90	Ovarian and breast cancers	Rapidly progressive cerebellar syndrome
Ma2 (and/or Ma)	>75	Testicular cancer, NSCLC	LE, diencephalitis, brainstem encephalitis
Tr (DNER)	90	Hodgkin lymphoma	Rapidly progressive cerebellar syndrome
KLHL11	80	Testicular cancer	Brainstem/cerebellar syndrome
AMPAR	>50	SCLC, malignant thymoma	LE
GABABR	>50	SCLC	LE (especially in elderly, smokers)
NMDAR	38	Ovarian teratomas	Anti-NMDAR encephalitis (more common in females aged 12–45)
CASPR2	50 (in Morvan syndrome)	Malignant thymoma	Morvan syndrome (peripheral nerve hyperexcitability, dysautonomia), LE
mGluR5	≈50	Hodgkin lymphoma	Encephalitis
P/Q VGCC	50 (LEMS); 90 (cerebellar)	SCLC	LEMS, rapidly progressive cerebellar syndrome

Abbreviations: ANNA = antineuronal nuclear antibody; CRMP5 = collapsin response-mediator protein 5; DNER = delta/notch-like epidermal growth factor—related receptor; EM = encephalomyelitis; KLHL11 = Kelch-like protein 11; LE = limbic encephalitis; LEMS = Lambert–Eaton myasthenic syndrome; MAP1B = microtubule-associated protein 1B; NMDAR = NMDA receptor; NSCLC = non-small-cell lung cancer; OMS = opsoclonus-myoclonus syndrome; PCA = Purkinje cell antibody; SCLC = small-cell lung cancer; SNN = sensory neuronopathy; SPS = stiff-person syndrome; AMPAR = α-amino-3-hydroxy-5-methyl-4-isoxazolepropionic acid receptor; GABABR = gamma-aminobutyric acid-b receptor; mGluR5 = metabotropic glutamate receptor type 5; VGCC = voltage-gated calcium channel.

## Data Availability

The original contributions presented in this study are included in the article. Further inquiries can be directed to the corresponding author.
